# Changing Preferences for a Cervical Cancer Screening Strategy: Moving Away from Annual Testing

**DOI:** 10.1089/whr.2022.0007

**Published:** 2022-08-04

**Authors:** Elizabeth Schrier, Hunter K. Holt, Miriam Kuppermann, George F. Sawaya

**Affiliations:** ^1^ School of Medicine, University of California, San Francisco, San Francisco, California, USA.; ^2^ Department of Family and Community Medicine, University of California, San Francisco, San Francisco, California, USA.; ^3^ Department of Family and Community Medicine, University of Illinois at Chicago, Chicago, Illinois, USA.; ^4^ Department of Obstetrics, Gynecology, and Reproductive Sciences, University of California, San Francisco, San Francisco, California, USA.; ^5^ Center for Healthcare Value, University of California, San Francisco, San Francisco, California, USA.

**Keywords:** patient preferences, cervical cancer screening, informed decision-making, patient education, high-value care

## Abstract

**Background:**

While annual cytology has not been recommended for many years, it remains many patients' preferred screening strategy for cervical cancer. Patient education and provider recommendations have been found effective in aligning professional society guidelines with patient preferences. We assessed whether an educational video with value elicitation exercises (utility assessments) changed screening strategy preferences among patients who had an initial preference for annual screening.

**Materials and Methods:**

We conducted an interventional study of English- or Spanish-speaking women 21–65 years of age, recruited from two women's health clinics in San Francisco, California (*n* = 262). Participants were asked about their preferred method of screening before viewing a 7-minute educational video and using a computerized tool that elicited values for 23 different health states related to cervical cancer screening. Directly afterward, they were again asked about their preferred screening strategy. Multivariable regression analysis was utilized to identify independent predictors of changing preferences.

**Results:**

Of 246 enrollees, 62.6% (154/246) had an initial preference for annual cytology; after viewing the video and completing the values elicitation exercises, about half (72/154, 47%) preferred a strategy other than annual screening. Having attended college and being screened every 3 to 5 years in the recent past were independent predictors of changing preferences away from annual screening. In sensitivity analyses, 53.2% of average-risk participants changed preferences away from annual cytology (*p* < 0.01).

**Conclusions:**

Viewing an educational video and conducting a series of value elicitation exercises were associated with a substantially decreased likelihood of preferring annual screening. These findings underscore the importance of patient-centered education to help support informed patient preferences.

## Introduction

Widespread screening has contributed to a substantial decline in cervical cancer incidence and mortality since the mid-20th century. Nonetheless, ∼13,800 cervical cancer diagnoses and 4,290 deaths occurred in the United States in 2021.^1^ Screening recommendations are intended to maximize the benefits of early cancer detection, while minimizing unnecessary treatments, psychosocial distress, life disruptions, and costs. In 2012, after decades of recommending annual screening, the US Preventive Services Task Force (USPSTF) and the American College of Obstetricians and Gynecologists (ACOG) endorsed cervical cytology every 3 years for individuals 21–65 years of age and cervical cytology and high-risk human papillomavirus (HPV) co-tests every 5 years for individuals 30–65 years of age.^2–4^

The American Cancer Society (and its partners) made a similar recommendation and specifically stated that average-risk “women should not be screened annually at any age by any method.” Average risk is defined as having no immunocompromising medical conditions (*e.g.*, HIV infection), having no exposure to diethylstilbestrol *in utero*, not being under active surveillance for a recent abnormality, and/or not having a diagnosis of cervical intraepithelial neoplasia (a precancerous lesion) within the last 25 years or a previous cervical cancer diagnosis.^5^ In 2018, the USPSTF also endorsed stand-alone HPV tests performed every 5 years for average-risk individuals with a cervix, 30–65 years of age, as an alternative to cervical cytology and co-testing.^6^

In order for new screening guidelines to be successfully adopted, they must be adequately communicated and reflect patient preferences and values.^7^ The USPSTF explicitly recommends that patients discuss their preferred screening strategy with their health care provider. Although annual cytology has not been recommended for many years, it continues to be a widely used screening strategy by providers and preferred by many patients.^8–12^ It is estimated that 60%–70% of patients believe they should be receiving annual cytology^9,13,14^ and over 50% prefer it to other screening strategies.^11,13^ Patients' fear of missing cancer—whether between extended screens or with HPV testing without cytology—is a common determinant of more intensive screening schedule preferences.^13,15^ A recent study in 2020 found that, while a third of patients were open to HPV screening every 5 years, only 11% preferred this option.^9^

Little is known about whether cervical cancer screening preferences reflect long-held beliefs or are based on patients' understanding of the screening process and differences between various screening tests. The objective of this study was to explore the effect of viewing an educational video and performing a series of value elicitation exercises on changing preferences for a screening strategy among patients with an initial preference for annual screening, including exploring factors associated with changing preferences. By achieving a better understanding of the characteristics and flexibility of screening preferences, we hoped to improve the shared decision-making process between patients and providers.

## Materials and Methods

### Study design

Our methods have previously been described.^16,17^ Briefly, a sociodemographically diverse group of English- or Spanish-speaking women, 21–65 years of age, was recruited from two women's health clinics in San Francisco, California, between 2014 and 2016 to take part in a study designed principally to measure patient preferences (utilities) to be used in the performance of cost-effectiveness analyses. Participants were first asked to complete a baseline questionnaire. They then took part in a 50-minute face-to-face interview that included viewing a 7-minute educational video.^18^

The video was developed as part of a cost-effectiveness study and described the differences between cytology, HPV testing, and colposcopy in terms of test accuracy (sensitivity and specificity). Cytology was described as having the lowest sensitivity (most missed disease) and the highest specificity (fewest “false alarms”), while HPV testing was described as having a higher sensitivity and lower specificity. Colposcopy was considered the reference standard with the highest sensitivity and specificity. The clinical procedures used to collect cytology HPV tests and to perform colposcopy were described with illustrations. The video purposely did not mention any screening strategy (*i.e.*, no particular test or tests at any specific periodicity).

Participants then performed a value elicitation exercise using a computerized tool. Values (utilities) for 23 different health states directly related to cervical cancer screening and treatment were elicited by employing the time-trade-off method.^19,20^ The health states included cytology, HPV, and colposcopy screening and varied from being told the results were normal, to receiving abnormal results and requiring further diagnostic testing, to being diagnosed with cervical cancer, requiring treatment, and ultimately dying from the disease. Each scenario included screening or treatment recommendations as well as common complications and possible emotions related to various health states. The educational video and computerized tool were available in English and in Spanish, as preferred by the participant.

As part of their baseline survey, the first 262 women enrolled in the study were asked to select an initial preferred screening strategy before viewing the educational video and completing the value elicitation exercises. These screening options were as follows: (1) once a year with cytology (a Pap smear) alone, (2) once every 3 years with cytology alone, (3) once every 3 years with an HPV test alone, (4) once every 3 years with both cytology and an HPV test, (5) once every 5 years with both cytology and an HPV test, and (6) once every 8 years with colposcopy alone.

Although colposcopy alone is not a strategy endorsed by any US guideline, we included it to be able to investigate preferences for, and the cost-effectiveness of, such a strategy in subsequent analyses; the strategy was modeled on current recommendations for colorectal cancer screening (annual fecal occult blood testing vs. less frequent colonoscopy). Participants also had the option of choosing “My health care provider told me that I don't need to be screened.” After viewing the educational video and completing the value elicitation exercises, participants were again asked to select a preferred screening strategy. The University of California, San Francisco, and Zuckerberg San Francisco General Hospital Institutional Review Boards approved this study, and written informed consent was obtained from all participants.

### Analysis

In the parent study, we reported the overall effect of the intervention on screening preferences among all participants before any exclusion was applied. In this analysis, we compared participants with initial preference for annual cytology to participants with all other initial screening preferences. We excluded participants with prior hysterectomy (*n* = 14) and those reporting “My health care provider told me that I don't need to be screened” (*n* = 2). We first compared demographic and clinical characteristics of those with an initial preference for annual cytology to those of all participants. Using the Stuart-Maxwell test, we then assessed changes in preferred screening strategy after the intervention for the entire study group (*n* = 246) and changes in preferred screening strategy among the subgroup of participants who initially preferred annual cytology (*n* = 154).

We used Pearson's chi squared testing in initial bivariate analyses, and then multivariable logistic regression to examine associations between preferences and preselected variables that we hypothesized may affect preferences: age, self-identified race/ethnicity, education, language, relationship status, frequency of previous cervical cancer screening, and previous HPV vaccination. Age was dichotomized to 21–34 years of age and 35 years and older, with the hypothesis that older women may be more accustomed to annual cytology and less likely to change their preference when compared to younger women. Self-identified race/ethnicity, education, and language were selected because of historical differential targeting of screening messages that have emphasized the importance of annual screening, observed disparities in uptake of cervical cancer screening services, and differing preferences for screening strategies.^13,21^

Relationship status was selected due to potential exposures to sexually transmissible infections such as HPV, which might vary by relationship status. Previous frequency of cervical cancer screening was included, given its possible effect on cervical cancer screening preferences.^13^ History of HPV vaccination was included as it was hypothesized that vaccinated women may be more amenable to less frequent screening. Because more intensive screening guidelines apply to women at higher-than-average cervical cancer risk, which may affect preferences, we grouped participants by risk. Higher-than-average risk was defined as having a history of HIV infection, cervical cancer, cervical dysplasia treatment, abnormal cytology, or colposcopy.

We conservatively included patients with a history of any abnormal cytology in the “higher-than-average risk” category because we were unable to assess the screening frequency recommended to these individuals before study enrollment; many may have completed post-treatment or post-colposcopy surveillance long ago and been advised to have less-than-annual screening as per management guidelines followed at the time of the study.^2^

### Missing data

Data were missing for two variables: HPV vaccination (*n* = 2) and frequency of prior screening (*n* = 5). Multiple imputation by chained equations was used to handle these missing data. To perform the imputation, all variables included in analysis model (including the outcome variable) were selected. Ordered logistic regression was used to impute previous cytology frequency and multinomial logistic regression was used to impute HPV vaccination history. One hundred imputations were performed to avoid producing a large Monte Carlo error.^22,23^

### Sensitivity/subgroup analysis

We performed one sensitivity analysis investigating outcomes in average-risk participants with an initial preference for annual cytology. Using the McNemar test, we assessed changes in preferred screening strategy after the intervention for this subgroup. We then performed multivariable logistic regression to examine associations between change in preference from annual cytology, adjusted for age, self-identified race/ethnicity, education, relationship status, language, frequency of previous cervical cancer screening, and previous HPV vaccination.

All analyses were performed with STATA 16, and we considered differences at the *p* < 0.05 level to be statistically significant.

## Results

### Participant demographics

Among all 246 participants, the mean age was 36.6 years (SD 10.4 years) and less than half (41.5%) self-identified as White (Table 1). Approximately 90% of these participants completed their interview in English. About half had graduated from college, and about two-thirds were married or in a relationship. Approximately 20% had received at least one dose of an HPV vaccine.

**Table 1.** Enrollee Characteristics: Comparing Characteristics of Those with an Initial Preference for Annual Pap Testing to Those Without This Initial Preference

Characteristic	Total (*n* = 246)	Initial preference for annual cytology (*n* = 154)	Initial preference not for annual cytology (*n* = 92)	OR (95% CI) for preferring annual cytology^a^	aOR (95 CI) for preferring annual cytology^b^
Age					
21–34 years	138 (56.1%)	77 (50.0%)	61 (66.30%)	1.00	1.00
35+ years	108 (43.9%)	77 (50.0%)	31 (33.70%)	1.97 (1.15–3.36)	1.95 (0.99–3.85)
Race/ethnicity					
White	102 (41.5%)	50 (32.5%)	52 (56.5%)	1.00	1.00
Black/African American	32 (13.0%)	25 (16.2%)	7 (7.6%)	3.71 (1.47–9.35)	1.40 (0.46–4.28)
Asian or Pacific Islander	35 (14.2%)	25 (16.2%)	10 (10.9%)	2.60 (1.13–5.96)	2.53 (1.00–6.42)
Latinx/Hispanic	45 (18.3%)	33 (21.4%)	12 (13.0%)	2.86 (1.33–6.15)	0.85 (0.30–2.36)
Other/Mixed	32 (13.0%)	21 (13.6%)	11 (12.0%)	1.99 (0.87–4.54)	1.45 (0.53–3.93)
Education					
Less than college	109 (44.3%)	86 (55.8%)	23 (25.0%)	1.00	1.00
College graduate or higher	137 (55.7%)	68 (44.2%)	69 (75.0%)	0.26 (0.15–0.47)	0.37 (0.17–0.80)
Relationship status					
Single	78 (31.7%)	57 (37.0%)	21 (22.8%)	1.00	1.00
In relationship or married	168 (68.3%)	97 (63.0%)	71 (77.2%)	0.50 (0.28–0.90)	0.93 (0.46–1.91)
Language					
English	226 (91.9%)	136 (88.3%)	90 (97.8%)	1.00	1.00
Spanish	20 (8.1%)	18 (11.7%)	2 (2.2%)	5.96 (1.35–26.29)	3.70 (0.65–20.87)
Frequency of prior screening					
At least every 1–2 years	183 (75.9%)	127 (85.2%)	56 (60.9%)	1.00	1.00
Every 3–5 years	49 (20.3%)	17 (11.4%)	32 (34.8%)	0.23 (0.12–0.46)	0.24 (0.11–0.51)
More than 5 years	9 (3.7%)	5 (3.4%)	4 (4.3%)	0.55 (0.14–2.13)	0.59 (0.13–2.70)
Prior HPV vaccination					
Yes	49 (20.1%)	24 (15.8%)	25 (27.2%)	1.00	1.00
No	162 (66.4%)	103 (67.8%)	59 (64.1%)	1.82 (0.95–3.47)	1.21 (0.54–2.71)
Do not know	33 (13.5%)	25 (16.4%)	8 (8.7%)	3.26 (1.23–8.62)	1.87 (0.60–5.92)
Risk level					
Average-risk	118 (48.0%)	62 (40.3%)	56 (60.9%)	1.00	1.00
Higher-than-average risk	128 (52.0%)	92 (59.7%)	36 (39.1%)	2.31 (1.36–3.91)	2.03 (1.10–3.72)

^a^ Bivariate logistic regression model.

^b^ Imputed logistic regression model adjusted for all variables in table.

aOR, adjusted odds ratio; CI, confidence interval; HPV, human papillomavirus; OR, odds ratio.

### Cervical cancer screening preferences

**FIG. 1.**
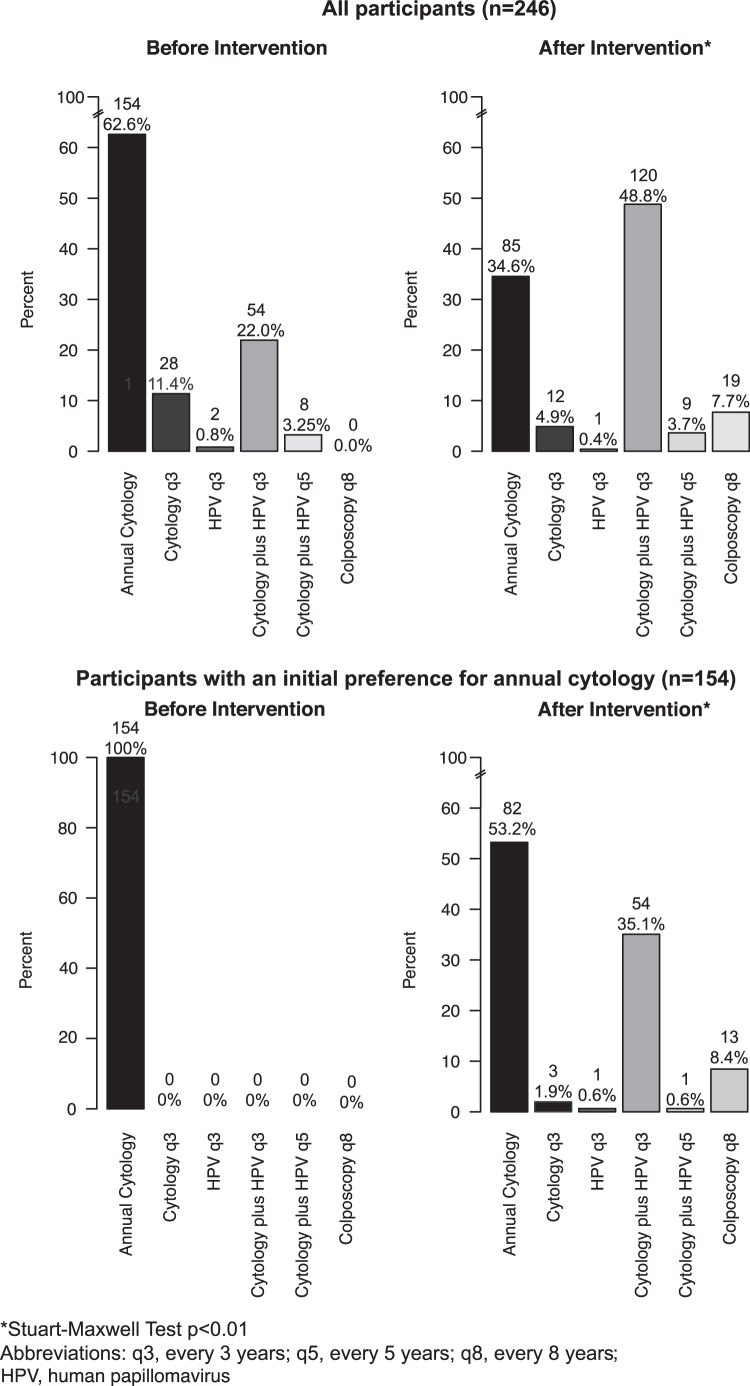
Cervical cancer screening preferences before and after the intervention, by initial preference.

Most participants (154/246, 62.6%) initially preferred annual cytology. In multivariable analyses, independent predictors for having an initial preference for annual cytology had less than a college degree and history of prior screening interval of at least 1–2 years, and belonged to a higher-than-average risk group (Table 1). Among all 246 participants, 109 (44.3%) changed their preferred screening strategy after viewing the educational video and completing the value elicitation exercises (Stuart-Maxwell *p* = <0.01, Fig. 1, top panel). In bivariate and multivariate analysis, no significant association between changes in preferences and the hypothesized predictors was detected (Table 2).

**Table 2.** Characteristics of Enrollees Associated with Changing Preferences, Stratified by Initial Preference

Characteristic	Any change of initial preference (*n* = 246)		Change away from an initial preference for annual cytology (*n* = 154)	
	OR (95% CI)^a^	aOR (95% CI)^b^	OR (95% CI)^a^	aOR (95% CI)^b^
Age				
21–34 years	1.00	1.00	1.00	1.00
35+ years	1.15 (0.70–1.92)	1.19 (0.66–2.13)	0.81 (0.43–1.53)	0.78 (0.36–1.66)
Race/ethnicity				
White	1.00	1.00	1.00	1.00
Black/African American	1.49 (0.67–3.32)	1.79 (0.70–4.60)	0.85 (0.33–2.23)	1.48 (0.46–4.78)
Asian or Pacific Islander	1.11 (0.51–2.40)	1.12 (0.50–2.50)	0.73 (0.28–1.90)	0.80 (0.27–2.32)
Latinx/Hispanic	0.88 (0.43–1.79)	1.13 (0.43–1.93)	0.53 (0.21–1.30)	1.41 (0.36–5.45)
Other/Mixed	1.03 (0.46–2.28)	1.08 (0.45–2.62)	1.02 (0.37–2.82)	1.20 (0.36–4.02)
Education				
Less than college	1.00	1.00	1.00	1.00
College graduate or higher	1.17 (0.70–1.94)	1.39 (0.71–2.72)	2.16 (1.13–4.13)	2.75 (1.11–6.81)
Relationship status				
Single	1.00	1.00	1.00	1.00
In relationship or married	0.83 (0.48–1.43)	0.85 (0.45–1.57)	1.08 (0.56–2.07)	0.84 (0.39–1.81)
Language				
English	1.00	1.00	1.00	1.00
Spanish	0.65 (0.25–1.70)	0.80 (0.22–2.79)	0.40 (0.13–1.17)	0.52 (0.11–2.44)
Frequency of prior screening				
At least every 1–2 years	1.00	1.00	1.00	1.00
Every 3–5 years	1.25 (0.66–2.35)	1.19 (0.61–2.31)	3.35 (1.11–10.08)	3.41 (1.04–11.20)
More than 5 years	2.81 (0.68–11.61)	2.49 (0.59–10.59)	2.09 (0.34–12.97)	2.84 (0.42–19.15)
Prior HPV vaccination				
Yes	1.00	1.00	1.00	1.00
No	1.31 (0.59–2.16)	1.04 (0.50–2.16)	0.84 (0.34–2.04)	0.84 (0.29–2.43)
Do not know	1.74 (0.71–4.24)	1.57 (0.59–4.20)	1.08 (0.35–3.32)	1.12 (0.31–3.99)
Risk level				
Average-risk	1.00	1.00	1.00	1.00
Higher-than-average risk	0.73 (0.44–1.21)	0.68 (0.40–1.17)	0.65 (0.34–1.24)	0.61 (0.30–1.24)

^a^ Bivariate Logistic Regression model.

^b^ Imputed logistic regression model adjusted for all variables in table.

Among the 154 participants with an initial preference for annual cytology, about half (72/154, 46.8%) preferred a strategy other than annual cytology after the intervention (Stuart-Maxwell *p* = <0.01, Fig. 1, bottom panel); the most common revised preferences were for cytology plus HPV testing (co-testing) every 3 years (54/154, 35.1%) and colposcopy every 8 years (13/154, 8.4%) (Fig. 1, bottom panel). In bivariate analysis, two factors were associated with changing the preferred strategy from annual cytology to any other strategy: being a college graduate (odds ratio [OR] 2.16 confidence interval [95% CI] 1.13–4.13) and having been screened every 3 to 5 years before study enrollment (OR 3.35 95% CI 1.11–10.08) (Table 2).

In multivariable analyses, being a college graduate (adjusted OR 2.75 95% CI 1.11–6.81) and having been screened every 3 to 5 years before enrollment (adjusted OR 3.41 95% CI 1.04–11.20) remained independent predictors of changing preferences away from annual screening.

Because we found that most of our participants were higher-than-average risk (128/246, 52.0%), a population for whom more frequent screening is often recommended, we performed a sensitivity analysis restricted to 62 average-risk participants who had an initial preference for annual cytology. In this group, 33 (53.2%) changed preferences away from annual cytology after the intervention (McNemar's *p* < 0.01). We identified no independent predictor of changing preferences in this group in multivariable analysis.

## Discussion

We found that viewing an educational video and conducting a series of value elicitation exercises were associated with a substantially decreased likelihood of preferring annual screening. That we found this effect among the subgroup of average-risk women with an initial preference for annual screening suggests that cervical cancer screening preferences can be changed to be more concordant with contemporary screening guidelines. These findings underscore the need for continued efforts to find ways to better align patient preferences with professional guidelines.

Our finding that participants with an initial preference for annual cytology and who reported being screened every 3 to 5 years before study enrollment were more likely to prefer less-than-annual screening after the intervention, suggests that the intervention was useful in making them more comfortable with less-than-annual screening. This finding points toward a role for continued education about cervical cancer screening to ensure patients understand the reasoning behind new guidelines such that they may feel more comfortable adopting them in health systems that no longer support annual screening.

Our findings are consistent with earlier studies that have found annual cytology remains the most frequently preferred cervical cancer screening strategy.^8,13^ After the intervention, the most commonly preferred cervical cancer screening strategy was cytology plus HPV testing every 3 years (120/246, 48.8%), a strategy that is not recommended. Few opted for screening strategies with intervals greater than 3 years (28/246, 11.4%) or with cytology or HPV testing alone (13/246, 5.3%), even though these strategies are most consistent with current guidelines.^5,7^

In fact, a minority of participants ultimately preferred a screening strategy endorsed by national guideline groups; in the overall group, only 10 participants changed to a recommended strategy: 4 chose cytology every 3 years and 6 chose cytology plus HPV testing every 5 years. Interestingly, while no participant initially preferred colposcopy every 8 years, 19 (13 of who had an initial preference for annual cytology) selected this as their preferred strategy following the intervention, suggesting that a subset of patients may select a more invasive screening test with the least likelihood of missing cervical disease performed at an extended interval. These findings might reflect a disparity between professional society recommendations and patient preferences.

Of note, our sample included many participants we considered to be at higher-than-average risk (128/246, 52.0%), who were more likely to have an initial preference for annual screening (92/128, 71.9%) compared to average-risk individuals (62/118, 52.5%). Nonetheless, our sensitivity analysis restricted to average-risk participants demonstrated a marked change in preferences away from annual testing in this group. Finally, our intervention did not explicitly state professional society recommendations, which we have previously shown to be effective at aligning patient preferences with recommendations in routine pelvic examinations.^24^

These findings do, however, rebut the notion that patients will always prefer more testing when given the opportunity and suggest that education is a potentially effective means of shaping preferences. Because our parent study indicated that annual cytology has the highest cost, but confers less health benefit than 12 other screening strategies, the findings in this study suggest that providing patients with more information about various screening tests may help align patient preferences with strategies toward high-value care. In addition, guideline groups may consider more focused attention on the preferences of the target screened population over the course of guideline development.

Our study highlights the need to educate patients about the reasoning behind changes in cervical cancer screening recommendations. Previous studies have shown that patients who are more knowledgeable about screening practices are more likely to accept extended screening intervals.^14,25^ Annual cytology, however, continues to be recommended by many providers, who express concern that extended screening intervals may reduce patient satisfaction, contraceptive provision, patient health and wellbeing, clinic volume, and financial reimbursement.^8,26,27^ These concerns reflect the often contradictory incentives placed on providers. Because previous studies have shown that patients are more willing to extend screening intervals if recommended by their provider,^9,13,15,28^ and provider-focused educational interventions have been found to be effective in aligning recommendations with updated guidelines,^29^ we believe that more provider education is warranted.

Our study has important limitations. Small sample sizes in some categories limited our ability to explore associations between preferences and patient characteristics in our logistic regression models. Women enrolled in our study were from a single geographic area, which may limit our findings' generalizability. While we enrolled a sociodemographically diverse group, individual racial/ethnic categories were too small to allow sufficient statistical power for subanalyses. Because our intervention included an educational video and computerized preference elicitation tool, it is unclear which component led to the changes we observed.

Our study did not include HPV testing alone every 5 years (a strategy currently endorsed by the USPSTF), as the study was conducted before the recommendation of this strategy. Future studies may enroll larger numbers of participants and use randomization to estimate the independent effect of an educational intervention on screening preferences. They may also focus on identifying the most salient components such that a more focused intervention may be developed and its efficacy evaluated. Our study's strengths include its novelty as one of the few in women's health to assess the effect of an educational and value elicitation intervention on preferences for differing cervical cancer screening strategies. We also enrolled a sociodemographically diverse group of participants with more than half identifying as people of color.

## Conclusions

While patient preferences for cervical cancer screening strategies vary, our study demonstrates that preferences can be influenced by in-depth information regarding the screening process and careful consideration of how patients value the potential processes and outcomes of care. Providing patients with information on the benefits and harms of different screening tests and an opportunity to clarify their values has the potential to align preferences with professional society recommendations. Promoting such patient education strategies may also lead to higher-value cervical cancer screening from the perspectives of society, the health care sector, and women.

## Abbreviations Used

ACOG: American College of Obstetricians and Gynecologists
CI: confidence interval
HPV: high-risk human papillomavirus
OR: odds ratio
USPSTF: US Preventive Services Task Force
